# Utility and significance of clinical risk factor scoring model in predicting central compartment lymph node metastasis (CLNM) in patients with papillary thyroid cancer (PTC)

**DOI:** 10.12669/pjms.38.1.4450

**Published:** 2022

**Authors:** Wei Shen, Xiao-jia Pan, Qing-huai Li

**Affiliations:** 1 Wei Shen, Department of Thyroid and Breast Surgery, The Second Hospital of Hebei Medical University, Shijiazhuang, Hebei 050000, China; 2 Xiao-jia Pan, Department of Thyroid and Breast Surgery, The Second Hospital of Hebei Medical University, Shijiazhuang, Hebei 050000, China; 3 Qing-huai Li, Department of Thyroid and Breast Surgery, The Second Hospital of Hebei Medical University, Shijiazhuang, Hebei 050000, China

**Keywords:** Central compartment lymph node, Lymph node metastasis, Thyroid cancer

## Abstract

**Objectives:**

To establish and discuss the significance of a clinical risk factor scoring model in predicting central compartment lymph node metastasis (CLNM) (level VI) in patients with papillary thyroid cancer (PTC).

**Methods:**

A retrospective analysis was performed on 412 patients who underwent surgical treatment for PTC who were admitted to the Second Hospital of Hebei Medical University between July 2016 and May 2017, with the patients being divided into a CLNM group and a non-metastasis (NM) group. Risk factors such as sex, age, tumor diameter, capsular invasion, multifocality, and tumor location were recorded for scoring via maximum likelihood estimation (MLE)-based discriminant analysis. The scoring model was used for prospective analysis of CLNM in another 104 patients. Besides, the discriminant function that was developed using the risk factors based on the retrospective data derived from the 412 patients was evaluated by plugging the retrospective data in for specified variables, with a higher score indicating a greater risk of developing CLNM. Clinical diagnosis of CLNM was based on postoperative paraffin section pathology, which was adopted as the criterion to assess discriminative accuracy in the prospective and retrospective groups.

**Results:**

The discriminative accuracy of the scoring model was 71.8% in the retrospective group and 72.2% in the prospective group.

**Conclusions:**

The scoring model enables simplified, quantitative analysis of CLNM in PTC patients. The scoring model has clinical significance in that it provides a basis for the choice of operation, personalized postoperative treatment, and prognosis of PTC.

## INTRODUCTION

Thyroid cancer (TC) is a common and frequently occurring disease in otorhinolaryngology – head and neck surgery, and papillary thyroid cancer (PTC) accounts for 80% to 90% of the overall incidence. In some PTC patients, cervical lymph node or distant metastasis is present at diagnosis. Metastasis does not necessarily first appear but is most likely to occur in the central compartment (level VI) of the affected side.^1^ Ultrasonography is the main imaging technique for the detection of thyroid and regional lymph nodes, which exhibits excellent sensitivity and accuracy in the differential diagnosis between benign and malignant thyroid nodules. Compared with lateral neck ultrasound, which is shown to have a sensitivity of up to 93.8%, ultrasonography of central compartment lymph nodes (CLNs) displays a relatively low diagnostic sensitivity (about 31.3%) due to anatomical and technical reasons.^2^ This study presented a maximum likelihood estimation (MLE)-based retrospective analysis of a group of PTC patients to calculate the scores of relevant risk factors for central compartment lymph node metastasis (CLNM), and the scoring criteria were also applied to the prospective screening for CLNM in another group of PTC patients, aiming to assess the significance of this risk factor scoring model as a clinical guidance tool in predicting CLNM in PTC patients.

## METHODS

### Ethical approval:

The study was approved by the Institutional Ethics Committee of The Second Hospital of Hebei Medical University on March 22, 2021(No.:GL2012039), and written informed consent was obtained from all participants.

### Materials:

*Inclusion criteria:* A patient was rendered eligible for the study if he/she (1) had never received any operations on the neck; (2) underwent central compartment lymph node dissection (CLND) of the affected side; (3) had complete medical records, including B-mode ultrasound diagnostic reports and postoperative paraffin section pathology reports.

*Exclusion criteria:* A patient was ineligible to participate in this study if he/she (1) had metastatic thyroid cancer; (2) had comorbid conditions such as anaplastic thyroid cancer or other types of thyroid cancer.

### Retrospective Data:

Clinical data were collected from 412 patients who were admitted to our department during May 2013 and July 2016 and confirmed to have PTC based on postoperative paraffin section histology. There were 90 males and 322 females, with the male-to-female ratio of 1:3.6. The PTC patients were 14 to 81 years old, with the mean age of 44.5 years. Tumor diameters ranged from 0.2 cm to 3.8 cm, with the mean diameter of 1.22 cm. Capsular invasion was observed in 81 patients, with the invasion rate of 19.7%. Papillary thyroid microcarcinoma (PTMC) occurred in 184 patients, accounting for 44.7% of the total PTC cases. CLNM was detected in 197 patients, with the metastatic rate of 47.8%.

### Prospective Data:

Clinical data were obtained from 104 patients who were admitted to Department of Breast and Thyroid Surgery,the Second Hospital of Hebei Medical University during July 2016 and May 2017 and diagnosed with PTC based on postoperative paraffin section pathology. These participants included 24 males and 80 females at the age between 20 and 73, with the mean age of 46.6 years. Tumor diameters varied from 0.3 cm to 3.8 cm, with the mean diameter of 1.19 cm. Capsular invasion was detected in 9 patients, representing an invasion rate of 8.7%. PTMC was detected in 48 patients, making up 46% of the total PTC cases. CLNM was seen in 41 patients, denoting a metastatic rate of 39.4%.

### Clinical observations:

Six clinical risk factors were applied to the screening for CLNM: (1) Sex: male or female; (2) age: <45 or ≥45 years old; (3) tumor diameter (TD): <0.7 cm, 0.7-1.0 cm, 1.0-1.5 cm, 1.5-2.0 cm, or ≥2.0 cm; (4) capsular invasion (CI): invasive (Y) or not (N); (5) multifocality: multifocal (Y) or unifocal (N); (6) tumor location (TL): upper pole (upper 1/3 of the thyroid gland), middle pole (middle 1/3 of the thyroid gland), lower pole (lower 1/3 of the thyroid gland), and isthmus. A frequency distribution was developed using the six clinical risk factors as enumeration data based on the retrospective data derived from the 412 PTC cases (Table-I).

**Table-I:** Frequency distribution of clinical risk factors for CLNM detection in the retrospective group (cases).

RF		CLNs	

		+	-
Sex	Female	144	178
	Male	53	37
Age	<45 yrs	111	95
	≥45 yrs	86	120
TD	<0.7 cm	18	74
	0.7-1.0 cm	37	55
	1.0-1.5 cm	53	62
	1.5-2.0 cm	35	16
	≥2.0 cm	54	8
CI	Y	61	20
	N	136	195
Multifocality	Y	13	9
	N	184	206
TL	Upper pole	26	48
	Middle pole	106	111
	Lower pole	57	45
	Isthmus	8	11

### Statistical Analysis:

The frequency of occurrence P(X_kj_/Y_g_) of each clinical risk factor was calculated using the data available in Table-I to determine the presence of metastasis in the central compartment, where Y represents the type of CLNs, with g =1 meaning positive (+) and g =2 negative (-) for metastatic lymph nodes, and X_kj_ (k =1, 2, 3, 4, 5, 6; j =1, 2) stands for the jth class of the kth risk factor. An MLE-based discriminant analysis of qualitative data was conducted to calculate the probability of CLNs being classified as g when a patient falls in the jth class of the kth risk factor: P_g_ =P(X_1j_ /Y_g_) ×P(X_2j_ /Y_g_) … ×P(X_6j_ /Y_g)_. Log probability can be calculated using the following equation: lgP_g_=lgP(X_1j_ /Y_g_) ×P(X_2j_ /Y_g_) … ×P(X_6j_ /Y_g_). The score of each risk factor can be obtained from the following equation:[lgP(X_kj_ /Y_g_) +1] (Table-II). The scoring formula is written as follows: S_g_ ={[lgP(X_1j_ /Y_g_) +1] +[lgP(X_2j_ /Y_g_) +1] …+[lgP(X_6j_ /Y_g_) +1]} ×10. Discriminant analysis was performed on the prospective group (n =104) and the retrospective group (n =412), respectively, using the above-mentioned scoring formula.

**Table-II:** Scores of the six clinical risk factors based on discriminant analysis.

RF		CLNs	
		+	-
Sex	Female	8.6	9.2
	Male	4.3	2.4
Age	<45 yrs	7.5	6.5
	≥45 yrs	6.4	7.5
TD	<0.7 cm	-0.4	5.4
	0.7-1.0 cm	2.7	4.1
	1.0-1.5 cm	4.3	4.6
	1.5-2.0 cm	2.5	-1.3
	≥2.0 cm	4.4	-4.3
CI	Y	4.9	-0.3
	N	8.4	9.6
Multifocality	Y	-1.8	-3.8
	N	9.7	9.8
TL	Upper pole	1.2	3.5
	Middle pole	7.3	7.1
	Lower pole	4.6	3.2
	Isthmus	-3.9	-2.9

### Operation methods and postoperative follow-up:

The retrospective and prospective groups consisted of 516 patients in total. All patients had received standard surgical treatment, including 214 cases of thyroid lobectomy with isthmusectomy and homolateral CLND, 218 cases of total thyroidectomy with homolateral CLND, and 84 cases of total thyroidectomy with bilateral CLND. Levothyroxine sodium tablets were administered orally after surgery, in combination with thyroid-stimulating hormone (TSH) suppression therapy. Thyroid function was tested a month after surgery to adjust the dose of levothyroxine sodium tablets according to the TSH level. During the adjustment period, thyroid function tests were taken every one and a half months. Thyroid and cervical lymph node ultrasound was performed six months after surgery. Subsequent reexaminations were recommended every six months, including physical checkup, thyroid function test, B-mode ultrasound, electrocardiogram (ECG), and chest X-ray. The patients were advised to seek prompt medical attention if any symptoms occurred. Except two patients who were lost to follow-up because they had changed their contact information without prior notice, all the other patients had completed follow-up for one to four years, with the mean follow-up period reaching 28.3 months. Postoperative hypocalcemia and hoarseness were observed in some cases, and full recovery was achieved within six months after surgery. No serious complications were detected.

## RESULTS

Scores of the six risk factors for CLNM detection are as shown in Table-II. The results of discriminant analysis showed that in the retrospective group, correct diagnoses were made in 129 positive (+) and 168 negative (-) cases of CLNM, with the coincidence rates of 65.5% and 78.1%, respectively, and the mean coincidence rate was 71.8% (Table-III); as to the prospective group, correct diagnoses were made in 28 positive (+) cases and 48 negative (-) cases of CLNM, with the coincidence rates of 68.2% and 76.2%, respectively, and the mean coincidence rate was 72.2% (Table-IV).

**Table-III:** Discriminative accuracy in the retrospective group (cases).

Final Diagnosis	Discriminant Result		Total	Coincidence Rate (%)

	+	-		
+	129	68	197	65.5
-	47	168	215	78.1
Mean	——	——	——	71.8

**Table-IV:** Discriminative accuracy in the prospective group (cases).

Final Diagnosis	Discriminant Result		Total	Coincidence Rate (%)

	+	-		
+	28	13	41	68.2
-	15	48	63	76.2
Mean	——	——	——	72.2

## DISCUSSION

PTC is a clinically common disease, with B-mode ultrasound as the mainstay of differential diagnosis between malignant and benign thyroid nodules. It was shown in Dan HJ et al.^3^ that high-frequency color Doppler ultrasound could produce a diagnostic accuracy of up to 87.64% in the diagnosis of PTMC. However, when applied to the diagnosis of CLNM, color ultrasound only has a sensitivity of 31.3%^2^, substantially lower than that in the diagnosis of lateral lymph node metastasis (up to 93.8%^4^). This is probably associated with the unique anatomical position (adjacent to the thyroid and the air-containing trachea) of the CLNs or other technical factors.

CLNM risk may differ by diverse factors, six of which were chosen for discriminant analysis: *(1) Sex:* male patients were at higher risk of lymph node metastasis than female patients^5^; among the 516 patients, 66 out of the 114 male patients were diagnosed with CLNM, and 172 out of the 402 female patients had CLNM, with the metastatic rates of 57.9% and 42.8%, respectively. *(2) Age:* According to the Chinese guidelines on tumor-node-metastasis (TNM) staging (2012 edition) and foreign studies, an age ≥45 is considered as a high-risk factor for thyroid cancer.^6^ Notably, it was reported in Ito et al.^7^ that a younger age entailed a higher metastatic rate, which was up to 50% in TC patients under 20. *(3) Tumor diameter*: In the NCCN clinical practice guidelines in oncology, a tumor diameter smaller than 1-cm was considered as a high-risk factor of neck lymph node metastasis^8^. PTC patients with a tumor diameter greater than 2 cm are shown to have higher rates of CLNM and lateral cervical lymph node metastasis (LCLNM) compared with those with a tumor diameter of or smaller than 2- cm.^9^ In some studies, the threshold tumor diameter is set as 0.5 cm or 0.6 cm to analyze CLNM in PTMC patients. In a preliminary study^10^, it was proposed that in PTMC, a tumor diameter of or greater than 0.7 cm had statistical significance in predicting CLNM. *(4) Capsular invasion*: Radowsky et al. viewed capsular invasion by tumor cells was a key risk factor for the prognosis of PTC patients.^11^ When capsular invasion occurs, the invasiveness of PTC cells increases, which is associated with the reduced inhibitory effect of the thyroid extracellular matrix (ECM) on lymph node metastasis in PTC, as well as the invasion of the thyroid’s compact lymphoreticular system by PTC cells.^12^ *(5) Multifocality*: Kuo et al.^13^ reported that compared with unifocal PTMC, multifocal PTMC exhibited a significant increase in the metastatic rate, consistent with the findings in their preliminary study. *(6) Tumor location:* Zhang et al.^14^ pointed out that when the foci occurred in the upper pole of the thyroid, there was an increased risk of LCLNM, but the risk of CLNM was reduced. Wang et al.^15^ found that tumors in the mid- and lower pole of the thyroid were more likely to induce CLNM. Based on the data given in Table-I, when the foci are present in the upper, middle, and lower poles and the isthmus, the CLNM rates are 35.1%, 48.8%, 55.9%, and 42.1%, respectively.

### Limitations of the study:

In this study, the MLE-based discriminant analysis enabled a detailed, quantitative diagnosis of CLNM in PTC patients, and the discriminative accuracy of the scoring model was up to 72%, surpassing that of B-mode ultrasound. Therefore, it is considered that this method can provide clinical guidance for predicting CLNM in PTC patients. Despite the extensive studies on risk factors for CLNM at home and abroad, little research has been done on particular individuals. Reportedly, CLNM is also associated with comorbidities like Hashimoto’s thyroiditis, as well as the TSH level, which is not considered in this study. Besides, this is not a multicenter study, and the choice of cases inevitably brings a bias to the study results. Although there are a few limitations, hopefully, this study can provide a basis for further studies to extend the knowledge in the field and improve the scoring model.

## CONCLUSIONS

Indications of prophylactic CLND for cN0 PTC patients are still controversial at home and abroad. The present study used retrospective data to obtain frequency distributions of clinical risk factors for PTC and carried out an MLE-based discriminant analysis of the prospective group; the discriminant analysis was evaluated using the retrospective data. With the discriminant rules remaining unchanged when the actual probability was converted to log probability, the risk factor scoring criteria were established accordingly. For example, if a 28-year-old male patient is diagnosed with unifocal PTC with a tumor diameter of 1.6 cm, and the tumor is located at the lower pole of the lateral thyroid without any signs of capsular invasion, his scores for the six risk factors are as follows: S_positive_ =37, S_negative_ =30.2, S_positive_ >S_negative_. The discriminant results show that this patient has CLNM.

### Authors’ Contributions:

**WS & Q-HL:** Designed this study and prepared this manuscript,and are responsible and accountable for the accuracy or integrity of the work

**X-JP:** Collected and analyzed clinical data

**Q-HL:** Significantly revised this manuscript.
